# Neurometabolic correlates of 6 and 16 weeks of treatment with risperidone in medication-naive first-episode psychosis patients

**DOI:** 10.1038/s41398-020-0700-6

**Published:** 2020-01-21

**Authors:** Badari Birur, Nina Vanessa Kraguljac, Lawrence VerHoef, Charity J. Morgan, Ripu Daman Jindal, Meredith Amanda Reid, Austin Luker, Adrienne Carol Lahti

**Affiliations:** 1 Department of Psychiatry and Behavioral Neurobiology, University of Alabama at Birmingham, Birmingham, AL USA; 2 Department of Neurology, University of Alabama at Birmingham, Birmingham, AL USA; 3 Department of Neurology, Birmingham VA Medical Center, Birmingham, AL USA; 4 MRI Research Center, Department of Electrical and Computer Engineering, Auburn University, Auburn, AL USA

**Keywords:** Neuroscience, Schizophrenia

## Abstract

Antipsychotic medications are the cornerstone of treatment in schizophrenia spectrum disorders. In first-episode psychosis, the recommended time for an antipsychotic medication trial is up to 16 weeks, but the biological correlates of shorter and longer antipsychotic treatment trials in these cohorts remain largely unknown. We enrolled 29 medication-naive first-episode patients (FEP) and 22 matched healthy controls (HC) in this magnetic resonance spectroscopy (MRS) study, examining the levels of combined glutamate and glutamine (commonly referred to as Glx) in the bilateral medial prefrontal cortex (MPFC) with a PRESS sequence (TR/TE = 2000/80 ms) before initiation of antipsychotic treatment, after 6 and 16 weeks of treatment with risperidone. Data were quantified in 18 HC and 20 FEP at baseline, for 19 HC and 15 FEP at week 6, and for 14 HC and 16 FEP at week 16. At baseline, none of the metabolites differed between groups. Metabolite levels did not change after 6 or 16 weeks of treatment in patients. Our data suggest that metabolite levels do not change after 6 or 16 weeks of treatment with risperidone in FEP. It is possible that our choice of sequence parameters and the limited sample size contributed to negative findings reported here. On the other hand, longer follow-up may be needed to detect treatment-related metabolic changes with MRS. In summary, our study adds to the efforts in better understanding glutamatergic neurometabolism in schizophrenia, especially as it relates to antipsychotic exposure.

## Introduction

Antipsychotic medications are the foundation of acute and maintenance treatment in schizophrenia spectrum disorders. In multi-episode schizophrenia, effectiveness of the medication is typically established within 2–6 weeks of treatment^1–5^. Recommendations for these timeframes as adequate duration of antipsychotic treatment trials are supported by meta-analyses demonstrating that the greatest fraction of antipsychotic response occurs within the first 2 weeks^6,7^, and that a substantial proportion of symptom improvement after 1 year has already been achieved within the first 4 weeks^7^.

In first-episode psychosis, Gallego and colleagues reported that the cumulative response rate increased from 32% after 6 weeks of treatment to 65.2% after 16 weeks of treatment in first-episode patients^8^, arguing that treatment trials in first-episode psychosis should last several months. Unfortunately, the underlying mechanisms and biological correlates of short and longer treatment trails in this patient cohort remain largely unknown.

One method to investigate biological signatures of antipsychotic treatment is magnetic resonance spectroscopy (MRS), a non-invasive imaging technique used to study chemical composition, energy metabolism, and neurometabolites in vivo^9^. In the context of psychosis, examination of glutamatergic measures are of particular interest for several reasons. First, N-methyl-D-aspartate (NMDA) receptor hypofunction is thought to be a core feature of the pathophysiology^10–14^, possibly resulting in downstream dopaminergic dysfunction^15–17^. Second, several groups, including ours, have reported alterations in glutamatergic measures including glutamate, glutamine, and glutamine + glutamate (Glx) in schizophrenia^18–24^. Third, a growing body of evidence suggests that antipsychotic treatment may decrease glutamatergic metabolite levels^22,25–30^, but also see a recent study by our group that did not find changes in glutamatergic metabolites in the anterior cingulate cortex and hippocampus after 6 weeks of antipsychotic treatment in patients with schizophrenia^31^.

The aim of our study was to examine longitudinally, neurometabolite levels in medication-naive first-episode psychosis (FEP) patients and changes after 6 and 16 weeks of treatment with risperidone, a commonly used second-generation antipsychotic medication. We chose the medial prefrontal cortex (MPFC) as our region of interest, because glutamatergic metabolites have been reported to be increased in this region in unmedicated, but not in medicated patients with schizophrenia^22,26–30,32–35^, suggesting that antipsychotic medications may affect glutamatergic measures in this area. Importantly, a recent study has reported that 4 weeks of treatment with risperidone resulted in a normalization of elevated MPFC Glx in medication-naive FEP^36^. Similarly, Egerton and colleagues reported a reduction in glutamate following treatment with amisulpride in FEP^26^.

Here, we scanned a group of antipsychotic-naive FEP, and then rescanned them after 6 and 16 weeks of treatment with risperidone to examine early and later medication effects on neurometabolite levels. We also scanned a group of matched healthy controls (HC) at the same intervals to assess the temporal stability of measurements. We hypothesized that^1^ Glx is increased, and that risperidone treatment^3^ decreases Glx. In an exploratory fashion, we also examined other commonly measured metabolites, including choline (Cho) and creatine (Cr), and investigated relationships between metabolite levels and clinical variables.

## Methods

Medication-naive FEP were recruited from the emergency room, inpatient units, and outpatient clinics at the University of Alabama at Birmingham (UAB). HC matched on age, sex, and parental occupation were recruited by advertisements. Approval for this study was obtained from the UAB Institutional Review Board and subjects provided written informed consent.

Diagnoses were established through consensus of two board certified psychiatrists (ACL and NVK) considering medical records and the Diagnostic Interview for Genetic Studies (DIGS)^37^ as available. The Brief Psychiatric Rating Scale (BPRS) and Repeatable Battery for the Assessment of Neuropsychological Status (RBANS) were used to assess symptom severity and cognition^38^.

Subjects were excluded if they had major neurological or medical conditions, history of head trauma with loss of consciousness, substance use disorders (excluding nicotine) within 6 months of imaging, were pregnant or breastfeeding, or had MRI contraindications. Those with a positive drug screen were excluded. HC with a personal or family history in a first-degree relative of a psychiatric disorder were excluded.

Subjects who were antipsychotic-naive were enrolled in a 16-week trial of risperidone using a flexible dosing regimen. As part of a larger multimodal neuroimaging study, MRS of the MPFC was done prior to initiation of treatment, after 6 weeks of treatment, and again after 16 weeks of treatment. Risperidone was prescribed by ACL and NVK; it was started at 1–2 mg and titrated in 1–2 mg increments; dosing was based on therapeutic and side effects. Use of concomitant medications after risperidone was started was permitted as clinically indicated. Compliance was monitored with pill counts at each visit.

To obtain normative data, matched HC were scanned at the same three time points as the FEP patients. Overall, we enrolled 29 FEP and 22 HC in this study.

### Image acquisition

Imaging was performed on a 3T head-only scanner (Magnetom Allegra, Siemens) using a circularly polarized transmit/receive head coil. A high-resolution structural scan was acquired (MPRAGE, TR/TE/TI = 2300/3.93/1100 ms, 1 mm isotropic voxels).

**Fig. 1:**
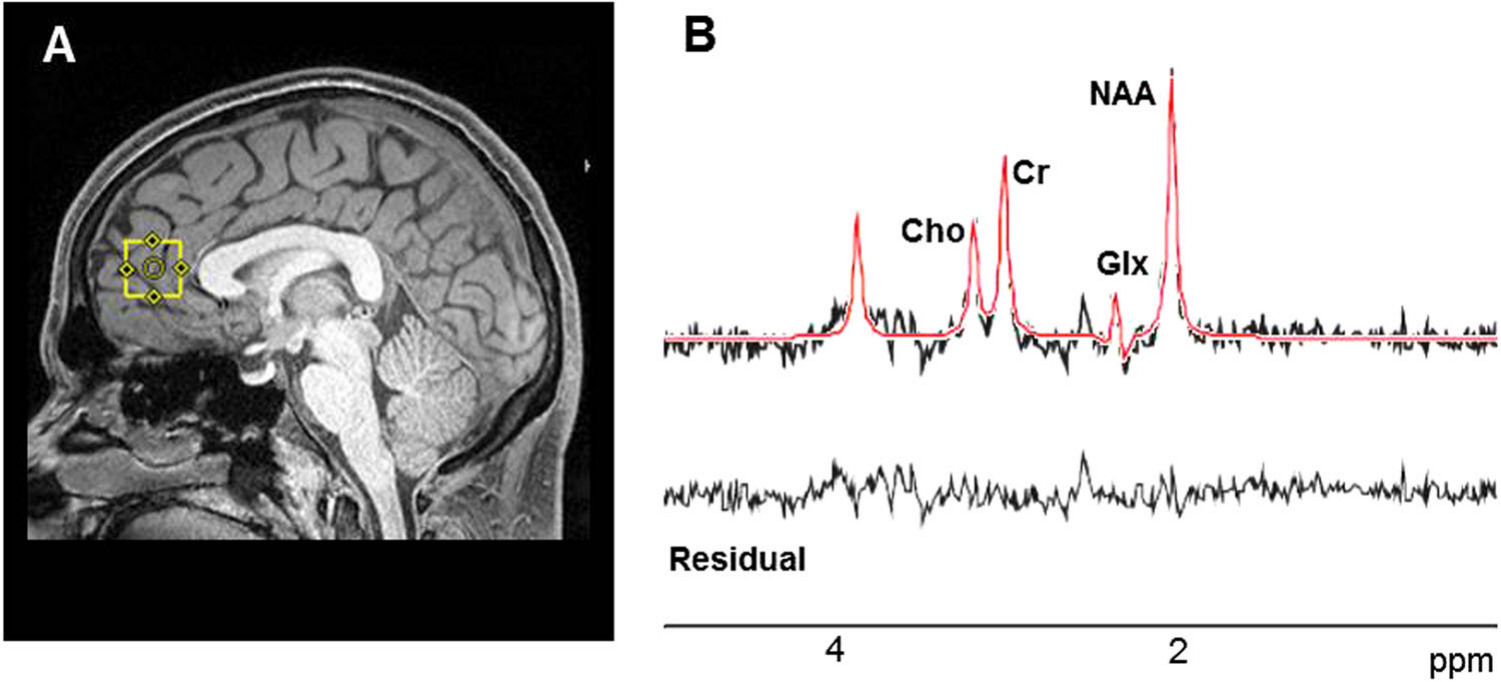
Illustration of voxel placement and example spectrum. **a** Voxel placement in the medial prefrontal cortex (2.0 × 2.0 × 2.0 cm). **b** Example spectra. The black line represents averaged spectra, the red line is an overlay of the spectral fit, the bottom line is the residual. Cho choline, Cr creatine, Glx glutamate + glutamine, NAA N-acetyl-aspartate, ppm parts per million.

A series of T1-weighted anatomical scans (TR/TE = 250/3.48 ms, 5-mm slice thickness) were acquired to aid placement of the bilateral MPFC (20 × 20 × 20 mm). Voxel placement for the second and third acquisition was guided by an image of the voxel placement during the first scan. After manual shimming, CHESS pulses were used to suppress the water signal. Spectra were acquired using a PRESS sequence (TR/TE = 2000/80 ms, to optimize the glutamate signal^39^ and minimize macromolecule contribution, 1200 Hz spectral bandwidth, 1024 points, 256 averages). Eight averages of unsuppressed water with the same sequence parameters were also acquired immediately afterwards to use as a reference. Examples of voxel placement and spectra are depicted in Fig. 1. To limit effects of nicotine intoxication or withdrawal, participants were allowed, but not encouraged, to smoke up to one hour before acquisition of images.

### MRS data processing

MRS data were quantified in the time domain with AMARES in jMRUI (version 5.2); prior knowledge derived from in vitro and in vivo metabolite spectra was included in the model, as reported elsewhere^40^. Exclusion criteria were (1) line width of the magnitude signal during manual shimming > 20 Hz at FWHM, (2) failure of fitting algorithm, and (3) Cramer–Rao lower bounds (CRLB) > 20%. Metabolites were quantified with respect to internal water (for comparison purposes also with respect to creatine). Structural scans were segmented into gray matter (GM), white matter (WM), and cerebrospinal fluid to calculate voxel tissue content, using in house Matlab codes.

### Statistical analyses

SAS version 9.4 (Cary, NC) was used for statistical analyses. Mixed effect repeated measures linear models were used to investigate the effects of group and time on neurometabolites. Voxel GM fraction (GM%/GM% + WM%^41^) was also included as a covariate in the model. Both a model assuming a common variance for HC and SZ, as well as a model that allowed for separate variances for HC and SZ were considered. Akaike’s information criterion was used to select the final model. A *p* value of < 0.05 was considered statistically significant.

In an exploratory fashion we also examined relationships between clinical characteristics (RBANS, BPRS, treatment response [defined as % decrease in BPRS total scores after 16 weeks of treatment], log transformed duration of untreated psychosis) and metabolites using partial correlations with age and gray matter fraction as covariates.

## Results

### Sample characteristics and demographics

Of the 29 FEP enrolled in this study, five dropped before the baseline scans were completed (four could not tolerate the scanning environment, one withdrew consent prior to scanning), one was excluded from all analyses due to gross anatomical abnormalities observed at the baseline anatomical scan, five dropped prior to the week 6 scan (one subject became pregnant, one moved out of state, one was lost to follow-up, one withdrew consent, one developed a neurological condition), and one subject did not tolerate the scan at week 16. In addition, MRS data were not obtained for three subjects at baseline, for three subjects at week 6, and for one subject at week 16 (either because of poor shimming quality or subject request to terminate the scan before obtaining the MPFC MRS scan) in this multimodal neuroimaging study.

To obtain normative data, 22 matched HC were scanned at the same three time points as the FEP patients with an average inter-scan interval of 49.5+/−13.6 days between the first and second scan (corresponding to baseline and week 6 scan in FEP), and 71.3+/−12.6 days between the second and third scan (corresponding to week 6 and week 16 scan in FEP). One HC was lost to follow-up after the first scan, and one was withdrawn from the study because they did not tolerate the MRI scan. In addition, MRS data were not obtained for two subjects and a water unsuppressed scan was not obtained for one subject at baseline, for two subjects at week 6, and for seven subjects at week 16 in this multimodal neuroimaging study.

In summary, quantifiable MRS data for the MPFC were available for 18 HC and 20 FEP at baseline, for 19 HC and 15 FEP at week 6, and for 14 HC and 16 FEP at week 16. For one FEP Cho and Cr values at week 6 were excluded because of our MRS data quality assessment criteria.

HC and FEP did not differ in gender, age, or parental socioeconomic status. As expected, RBANS scores were significantly lower in FEP compared with HC (Table 1).

**Table 1:** Demographics and clinical measures^a^.

	HC (*n* = 21)	FEP (*n* = 22)	*t*/*X*^2^	*p* value
Gender (% male)	62	64	0.01	0.91
Age	22.95 (5.01)	22.73 (5.52)	0.14	0.89
Parental occupation^b^	3.95 (3.15)	4.95 (5.00)	−0.78	0.44
Smoking status (% smoker)	0	32	7.98	<0.01
Diagnosis				
Schizophrenia		15		
Schizoaffective disorder		5		
Unspecified psychosis		2		
Duration of untreated psychosis^c^ (mean; median; [range] in weeks)		124.55; 45; [1–626]		
Risperidone dose week 6 (mean; median; [range] in mg)		3.61; 4; [1–8]		
Risperidone dose at week 16 (mean, SD; median; [range] in mg)		4.65; 4; [0–8]		
BPRS^d^				
Baseline				
Total		50.91 (9.41)		
Positive		11.55 (3.42)		
Negative		7.41 (3.57)		
Week 6				
Total		34.00 (10.88)		
Positive		5.67 (2.61)		
Negative		6.17 (2.83)		
Week 16				
Total		34.44 (11.44)		
Positive		5.44 (2.9)		
Negative		6.94 (2.82)		
RBANS^e^				
Total index	91.14 (13.32)	70.42 (14.61)	4.69	<0.01
Immediate memory	100.67 (15.57)	78.95 (16.7)	4.26	<0.01
Visuospatial	85.62 (13.67)	74.58 (15.21)	2.42	0.02
Language	94.81 (17.06)	81.79 (12.49)	2.73	<0.01
Attention span	94.62 (19.05)	75.16 (18.97)	3.23	<0.01
Delayed memory	91.81 (8.37)	71.53 (20.14)	4.23	<0.01

*FEP* first-episode psychosis, *HC* healthy control.

^a^Mean (SD) unless indicated otherwise.

^b^Ranks determined from Diagnostic Interview for Genetic Studies (1–18 scale); higher rank (lower numerical value) corresponds to higher socioeconomic status.

^c^Data on duration of untreated psychosis were not available for two patients.

^d^Brief Psychiatric Rating Scale (1–7 scale); positive (conceptual disorganization, hallucinatory behavior, and unusual thought content); negative (emotional withdrawal, motor retardation, and blunted affect).

^d^Repeatable Battery for the Assessment of Neuropsychological Status, data not available for three FEP.

### MRS measurements

**Fig. 2:**
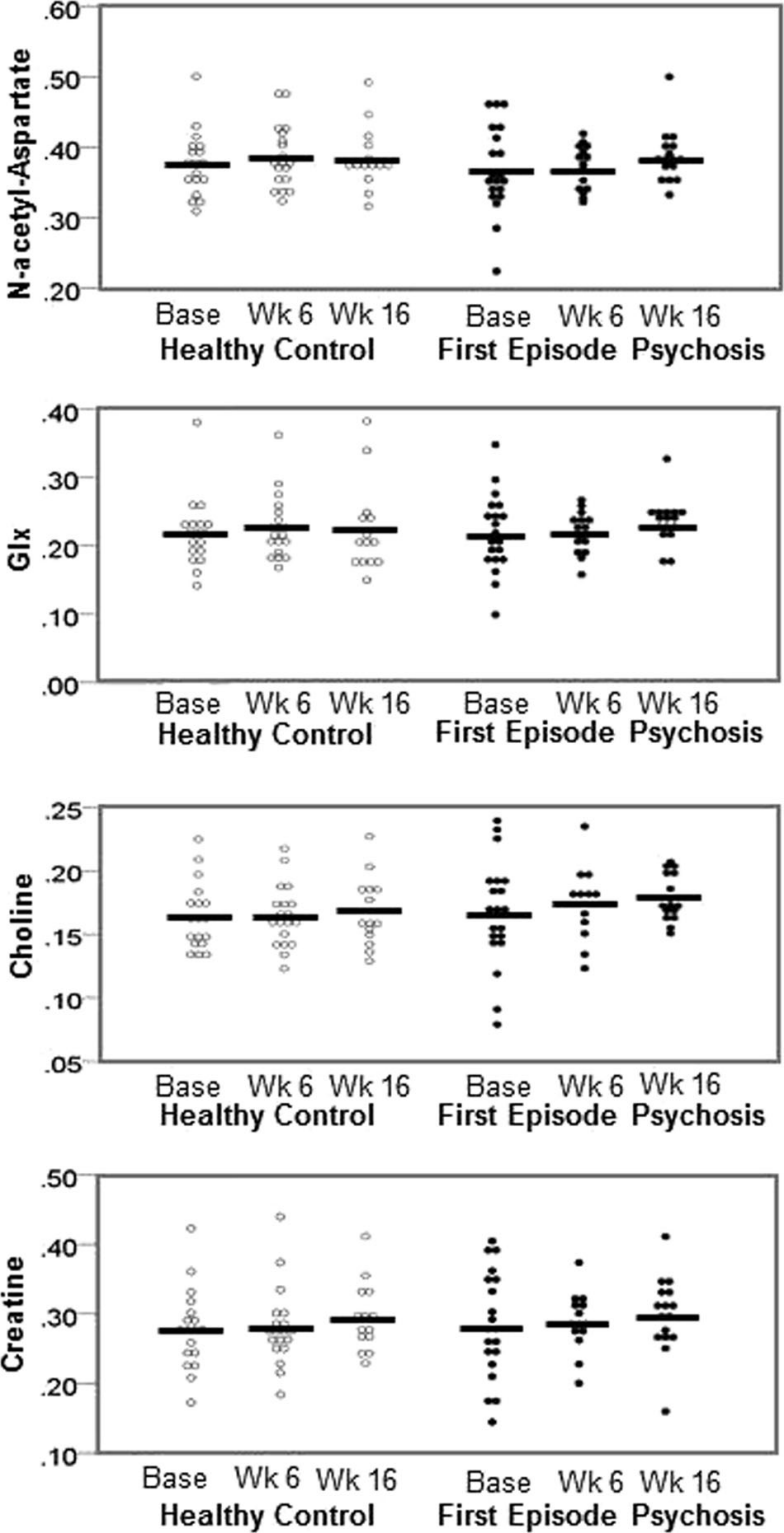
Neurometabolite measurements. Dots represent individual measurements, lines represent group means. Glx, glutamate + glutamine; base, baseline measurements; wk 6; measurements at week 6; wk 16, measurements at week 16.

Of the models for mixed effects repeated measures considered, the model assuming a common variance for HC and FEP had the best fit. After controlling for time and GM fraction, no significant differences in metabolites between groups were found, no group by time interaction was present (*p* > 0.2 for all; Table 2, Fig. 2). We also found no differences between metabolites when using creatine instead of internal water as a reference (Table 3).

**Table 2:** Neurometabolites, spectral quality, and tissue fraction.

	HC	FEP	F/*t*	*p*
*Neurometabolites*^a^				
Baseline				
NAA	0.38 (0.05)	0.37 (0.06)	0.29	0.77
Glx	0.22 (0.05)	0.22 (0.06)	0.08	0.94
Cho	0.16 (0.03)	0.17 (0.04)	−0.1	0.92
Cr	0.28 (0.06)	0.28 (0.08)	−0.25	0.80
Week 6				
NAA	0.39 (0.04)	0.37 (0.03)	1.05	0.30
Glx	0.23 (0.05)	0.22 (0.03)	0.66	0.52
Cho	0.16 (0.02)	0.17 (0.03)	−0.94	0.35
Cr	0.28 (0.06)	0.29 (0.04)	−0.43	0.67
Week 16				
NAA	0.39 (0.04)	0.39 (0.04)	−0.18	0.86
Glx	0.22 (0.07)	0.23 (0.03)	−0.53	0.60
Cho	0.17 (0.03)	0.18 (0.02)	−1.19	0.24
Cr	0.29 (0.05)	0.30 (0.06)	−0.23	0.82
*Spectral quality indices*				
Baseline				
NAA CRLB	1.50 (0.20)	1.70 (0.44)	−1.88	0.07
Glx CRLB	9.16 (1.70)	10.44 (3.07)	−1.61	0.12
Cho CRLB	2.77 (0.45)	3.24 (1.41)	−1.40	0.18
Cr CRLB	1.77 (0.28)	2.01 (0.71)	−1.31	0.20
FWHM	6.46 (2.16)	6.86 (1.59)	−0.66	0.52
SNR	15.18 (2.31)	13.72 (3.73)	−1.46	0.15
Week 6				
NAA CRLB	1.54 (0.30)	1.55 (0.22)	−0.21	0.84
Glx CRLB	10.16 (3.47)	9.02 (1.86)	1.21	0.24
Cho CRLB	2.87 (0.55)	2.68 (0.34)	1.12	0.27
Cr CRLB	1.84 (0.40)	1.88 (0.63)	−0.27	0.79
FWHM	7.60 (1.96)	6.49 (1.00)	2.15	0.04
SNR	16.09 (2.35)	14.37 (1.42)	2.67	0.01
Week 16				
NAA CRLB	1.54 (0.23)	1.56 (0.27)	−0.27	0.79
Glx CRLB	10.90 (3.56)	9.81 (2.40)	1.00	0.33
Cho CRLB	2.83 (0.30)	2.95 (1.25)	−0.34	0.73
Cr CRLB	1.79 (0.24)	2.04 (1.46)	−0.64	0.53
FWHM	7.99 (2.55)	7.50 (1.63)	0.63	0.53
SNR	16.09 (3.17)	15.39 (2.01)	0.74	0.47
*Voxel tissue fraction*				
Baseline				
Gray matter (%)	74.35 (5.59)	75.88 (3.88)	−1.01	0.32
White matter (%)	11.08 (7.71)	9.74 (3.49)	0.71	0.48
CSF (%)	14.78 (3.17)	13.85 (2.90)	0.97	0.34
Week 6				
Gray matter (%)	76.25 (2.81)	75.45 (2.84)	0.84	0.41
White matter (%)	9.07 (2.57)	9.16 (2.36)	−0.11	0.91
CSF (%)	14.82 (2.72)	15.45 (3.07)	−0.64	0.53
Week 16				
Gray matter (%)	75.30 (2.95)	76.18 (4.05)	−0.69	0.49
White matter (%)	10.58 (3.24)	9.07 (2.38)	1.52	0.14
CSF (%)	14.00 (2.15)	14.21 (3.59)	−0.23	0.82

*HC* healthy control, *FEP* first-episode psychosis patient, *NAA* N-acetyl-aspartate, *Glx* glutamate + glutamine, *Cho* choline, *Cr* creatine, *CRLB* Cramer–Rao lower bounds, *FWHM* full width half maximum, *SNR* signal-to-noise ratio, *CSF* cerebrospinal fluid.

^a^Institutional units.

**Table 3:** Neurometabolite concentrations, with creatine as reference.

	HC	FEP	*t*	*p*
*Neurometabolites*^a^				
Baseline				
NAA/Cr	1.55 (0.28)	1.48 (0.25)	0.72	0.48
Glx/Cr	0.84 (0.16)	0.81 (0.11)	0.70	0.49
Cho/Cr	0.73 (0.17)	0.74 (0.10)	−0.14	0.89
Week 6				
NAA/Cr	1.57 (0.22)	1.49 (0.26)	0.87	0.39
Glx/Cr	0.87 (0.10)	0.83 (0.13)	0.96	0.35
Cho/Cr	0.72 (0.15)	0.73 (0.09)	−0.04	0.97
Week 16				
NAA/Cr	1.48 (0.11)	1.41 (0.13)	1.48	0.15
Glx/Cr	0.80 (0.10)	0.81 (0.09)	−0.30	0.77
Cho/Cr	0.70 (0.08)	0.71 (0.08)	−0.43	0.67

*HC* healthy control, *FEP* first-episode psychosis patient, *NAA* N-acetyl-aspartate, *Glx* glutamate + glutamine, *Cho* choline, *Cr* creatine.

We found no correlations between neurometabolite levels and BPRS scores, RBANS scores, or duration of untreated psychosis.

## Discussion

Here we conducted a longitudinal MRS study prospectively examining the effects of antipsychotic treatment on glutamatergic measures in the MPFC in a group of medication-naive FEP patients. We did not find any baseline metabolite alterations in patients compared with HC. Interestingly, metabolite levels were not affected by medications both after 6 and 16 weeks of treatment with risperidone.

We found no increase in Glx in medication-naive FEP, which stands in contrast with prior findings by Kegeles et al. who showed elevated GABA and Glx in the MPFC in unmedicated schizophrenia patients^22^, and Chen et al. who reported elevated glutamate and reduced glutamine in the ventromedial prefrontal cortex in FEP who had been off antipsychotic medications for at least 2 weeks^23^. Examining glutamatergic measures in the MPFC across illness stages, Natsubori and colleagues reported no alterations in Glx in the MPFC in subjects at ultrahigh risk for schizophrenia or FEP patients with no more than 16 weeks of cumulative antipsychotic medication exposure, but did report a decrease in Glx in chronic schizophrenia patients^42^. In contrast, Wang and colleagues did not find alterations in Glx in ultrahigh risk subjects, but reported reduced MPFC Glx in a small group of medication-naive FEP^43^. It stands to reason that illness stage could, at least in part, account for discrepancies in findings. Furthermore, age effects and effects of prior medication exposure in studies before undergoing a medication washout may contribute to discrepancies in findings, where patients in early illness stages may be more prone to glutamatergic elevations than those in the chronic stages^44^.

In a similarly designed study, de la Fuente-Sandoval and colleagues enrolled 28 antipsychotic medication-naive FEP patients and assessed MPFC Glx before and after 4 weeks of treatment with risperidone^36^. In contrast to us, they report that elevated Glx was seen in patients at baseline, but no longer was present after treatment. Interestingly, the duration of untreated psychosis in this group was 52 weeks on average (only two subjects had experienced psychotic symptoms for more than 2 years), while in our sample it was ~2.4 years on average. Furthermore, the mean daily dose of risperidone in this sample was about 25% lower than in our sample, suggesting that the patients in our sample had an overall higher psychosis burden. It is possible that these important clinical differences could have contributed to discrepancies in findings. Future studies investigating the impact of duration of untreated psychosis on glutamatergic indices will be informative in this regard.

Another consideration are differences of acquisition techniques across various studies. While we used PRESS, Kegeles and colleagues used MEGA-PRESS to quantify Glx. A technical report comparing these acquisition sequences recently reported that spectral values for Glx acquired with a TE = 80 ms PRESS sequence correspond to the off-resonance but not the difference spectra acquired with a MEGA-PRESS sequence in healthy volunteers and medicated FEP subjects^45^. However, as the authors underscore, it remains an open question whether glutamatergic values from PRESS spectra are more or less valid than glutamatergic values from MEGA-PRESS difference spectra. A number of studies relied on shorter TE and spectral fitting to derive the levels of glutamatergic compounds that may explain some of the discrepancies. Last, glutamatergic alterations may vary depending on the region of interest studied, with most studies targeting the frontal regions, specifically the MPFC reporting elevations in glutamatergic compounds, while many other studies targeting more dorsal brain regions, e.g., the dorsal anterior cingulate cortex, found no elevations^46^.

Contrary to our hypothesis, we observed no decrease in Glx after 6 or 16 weeks of treatment with risperidone. The lack of changes in metabolite levels stands in contrast with a literature review suggesting that antipsychotic medications may affect glutamate levels, with an estimated 6.5% reduction in Glx across brain regions^27^. However, several studies report no antipsychotic induced changes^31^, or regionally selective reductions^29^ in glutamatergic measurements. It is possible that variable antipsychotic medications have diverse effects on the glutamate system. Investigating differential drug effects on brain metabolite levels in an animal model, McLoughlin and colleagues report antipsychotic induced reductions of glutamate in the frontal cortex only with clozapine and olanzapine, but not risperidone, haloperidol, or aripiprazole^47^. This is further supported by experiments with the NMDA receptor blocker ketamine, a popular pharmacological model of psychosis^11,12,48–53^, showing that pretreatment with high dose olanzapine, but not risperidone attenuates ketamine induced alterations in brain metabolism^54^.

### Strengths and limitations

Our findings have to be interpreted in context of a number of strengths and limitations. We enrolled exclusively medication-naive FEP and a group of HC matched on key demographic characteristics in this prospective longitudinal trial. Even though smoking rates differed between groups, metabolites did not. To minimize variance in the data, we used a single antipsychotic medication. We assessed adherence using pill counts and self-report, but future studies would benefit from monitoring risperidone blood levels to confirm compliance. Because it is ethically not permissible to withhold known effective medications from patients, we did not include a placebo group. On a technical note, we used a relatively long TE of 80 ms for MRS in an effort to optimize the glutamate signal^39^ and minimize macromolecule contribution, but this imparts appreciable J-modulation and T2 relaxation effects on the spectrum, and the water signal is highly T2 weighted and very sensitive to cerebrospinal fluid contamination, as well as partial volume effects. Last, it is possible that we did not have sufficient power to detect group differences in Glx or changes in Glx with antipsychotic treatment, as prior studies have suggested that at least 24 subjects in each group may be necessary to detect a reduction in Glx with antipsychotic treatment with 80% power^27^. The limited sample size reflects the difficulty in recruiting this patient population. Because of this, we were unable to dichotomize patients into good and poor treatment responders to determine if reductions in Glx are more likely in those with symptomatic improvement as recently reported by Merritt and colleagues^55^.

## Conclusions

The underlying mechanisms and biological correlates of short and longer treatment trails remain largely unknown, and it is possible that longer treatment durations may be needed to detect treatment-related changes in neurometabolites. In this prospective MRS study, we examined Glx before treatment was started, after 6 and again after 16 weeks of treatment, but failed to detect changes in metabolite levels.

In summary, our study adds to the efforts in better understanding glutamatergic neurometabolism in schizophrenia, especially as it relates to antipsychotic exposure. It will be critically important to systematically investigate glutamatergic measurements across various areas of the brain, different illness stages, and different medication statuses to improve our mechanistic understanding and facilitate biomarker development.
